# Immunological, hematological, biochemical, and histopathological studies on cows naturally infected with lumpy skin disease

**DOI:** 10.14202/vetworld.2015.1131-1136

**Published:** 2015-09-23

**Authors:** Ahmed N. F. Neamat-Allah

**Affiliations:** Department of Clinical Pathology, Faculty of Veterinary Medicine, Zagazig University, 1 Alzeraa Street, Postal Code 44511, Zagazig City, Sharkia Province, Egypt

**Keywords:** biochemistry, cows, hematology, histopathology, lumpy skin disease, virus

## Abstract

**Aim:**

Lumpy skin disease (LSD) is an infectious viral disease of cattle caused by LSD virus (LSDV) of the family Poxviridae characterized by skin nodules covering all parts of the body. There are many aspects of LSD remaining unknown, thus immunological, hematological, and biochemical parameters were estimated.

**Materials and Methods:**

During an outbreak of LSD in Sharkia governorate from Egypt, 211 cows aging (2-4 years) were examined clinically for the presence of LSD lesions during the period from July to November 2014. A total of 134 cows from those showed lesions suspected to be LSD.

**Results:**

Recorded clinical signs were pyrexia with the development of skin nodules of varying sizes which ranged from a few to several hundred sometimes coalesced together. Enlargements of the peripheral lymph nodes. Intracytoplasmic inclusion bodies were noticed in the histopathological examination. Immunological studies revealed a significant decrease of lymphocyte transformation rate, phagocytic % and killing % which was marked within 2 weeks postinfection. LSD resulted in non-significant in hemogram in 1^st^-2^nd^ day post-infection while a macrocytic hypochromic anemia within 10-14^th^ days post-infection. Leucopenia and lymphopenia were recorded 1^st^-2^nd^ day post-infection while at 10-14^th^ showed granulocytic leucocytosis. Biochemical analysis revealed hypoproteinemia, hypoalbuminemia, and hyperglobulinemia especially gamma globulins. There were a significant increase in serum alanine aminotransferase, aspartate aminotransferase activities, creatinine level, blood urea nitrogen and creatine phosphokinase

**Conclusion:**

LSDV infected cows in early stages revealed leucopenia. Immunosuppressive effect was pronounced later. In late stage revealed hemolytic anemia, leucocytosis and increase of serum CK, which could aid in diagnosis. Disturbance in liver and kidney function tests have been occurred.

## Introduction

Lumpy skin disease (LSD) is an infectious viral disease of cattle caused by LSD virus (LSDV) of the family *Poxviridae* virus known as (Neethling virus) characterized by fever and sudden eruption of multiple skin nodules covering all parts of the body [1]. LSD was first seen as an epidemic in Zambia in 1929, and since then has spread and affected cattle throughout Africa, including the countries of South Africa, Egypt, and Sudan [2]. Severe cyclic outbreaks continue to occur in Egypt during 1989, 2006 and 2011 were recorded in several Egyptian governorates, and then incursion of LSD was reported for the first time in Iraq and Turkey in 2013, indicating that the disease has a potential risk for further spread to the European Union and Caucasus Region, as well as to Asia [3-5]. The disease listed by OIE [6] in “List A” due to its rapid spread and severe economic losses, such as decrease in milk production and weight gain, mastitis, infertility, and death [7,8].

LSDV is primarily transmitted by biting insects. The virus have been isolated from mosquitoes of the genera *Aedes* and *Culex* and *Ixodid* ticks during some outbreaks [9] while direct contact between animals is not likely to be a significant source of spread of LSDV [4].

Despite of LSD could be diagnosed using serological and molecular techniques [10,11], histopathological examination remain an important tool to exclude viral, bacterial, or fungal causes development of skin nodular lesions in clinical cases and characteristic cytopathic effects (ballooning degeneration of squamous epithelial cells and eosinophilic intracytoplasmic inclusion bodies) in cases of LSD are well documented [12,13], but there are many aspects of LSD that remain unknown, so the immunological, hematological, and biochemical parameters of LSDV naturally infected cows were estimated in this study.

## Materials and Methods

### Ethical approval

Ethical approval from the Egyptian Veterinary Medicine Authority was obtained.

### Animal

During an outbreak of LSD in Sharkia governorate in Egypt, 211 cows aging (2-4 years) were examined clinically for the presence of LSD lesions during the period from July to November 2014. A total of 134 cows from those showed lesions suspected to be LSD. The animals were feverish, had multiple skin nodules and enlargement of superficial lymph nodes typical of LSD. Treatment was including washing by water, and dressing by povidone iodine and diclofenac sodium with oxytetracycline 20% long acting, as antipyretic administration and broad spectrum antibiotics. Another 10 healthy cows were used like normal control.

### Blood sampling

Blood was collected by puncture of the jugular vein from cows into three samples. The first blood samples were taken in heparinized tubes for immunological studies. The second blood samples were taken into ethylenediaminetetraacetic (EDTA) tubes for hematological analysis. The third blood samples were taken in a sterile test tube for separation of serum that was used for biochemical measurements. Samples were collected at (1^st^-2^nd^ days) and (10-14^th^) days post appearance of skin nodules.

### Immunological studies

A lymphocytic transformation assay using 3-(4,5-dimethylthiazol-2-7l)-2, 5-diphenyl 2H tetrazolium bromide was used with 2348-71-2which is a methyl tetrazolium dye staining procedure [14]. Blood samples were collected in heparinized tubes and used to prepare leucocyte for bacterial phagocytic activity and killing power [15].

### Hematological examination

Complete blood count was evaluated in an automatic cell counter (Hospitex Hemascreen 18, Italy).

### Biochemical examination

Protein electrophoresis was quantitatively measured [16], while creatine phosphokinase (CK-MM) was measured in full automated biochemistry analyzer (Chemray 240. USSR). Serum activities of alanine and aspartate aminotransferase (ALT and AST) were determined [17], serum creatinine and blood urea nitrogen levels were estimated [18,19].

### Histopathological studies

Selected skin nodules from affected animals were surgically removed after local anesthesia then fixed in 10% buffered neutral formalin solution, dehydrated in gradual ethanol (70-100%), cleared in xylene, and embedded in paraffin. Five micron thickness paraffin sections were prepared and then routinely stained with hematoxylin and eosin (H and E) dyes. The sections were mounted with Canada balsam and covered with the cover slide to be ready for histopathological examination [20].

### Statistical analysis

Data obtained from this investigation were statistically analyzed using the one-way analysis of variance using SPSS 16.0 for windows [21]. Means in the same row followed by different letters were significantly different, and the highest value was represented with the letter (a).

## Results and Discussion

**Figure-1:**
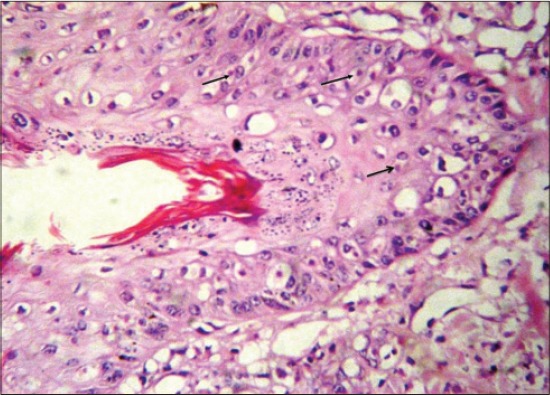
Lumpy skin disease, skin of cow showing ballooning and degeneration of prickle cell layer with scattered intracytoplasmic inclusion bodies (arrows), H and E, ×1200.

**Figure-2:**
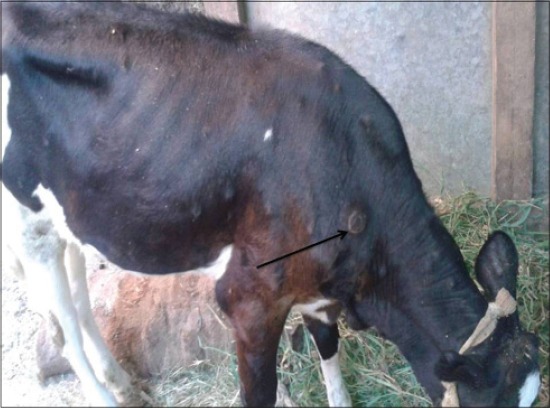
Lumpy skin disease in cow showing few skin nodules.

**Figure-3:**
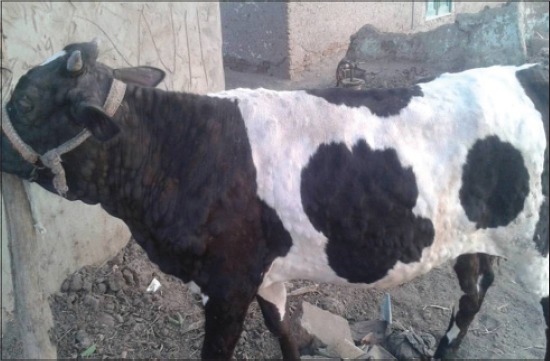
Lumpy skin disease in cow showing skin nodules cover all body parts.

**Figure-4:**
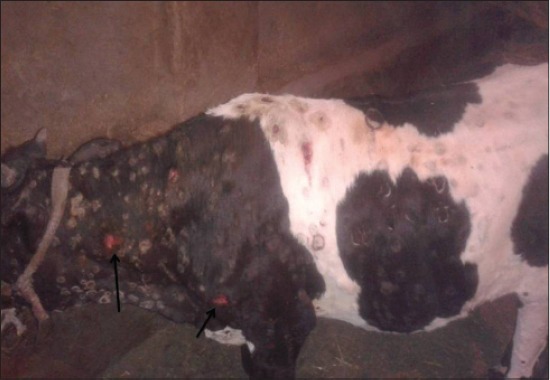
Lumpy skin disease in cow showing skin nodules leaving ulcer (14 days post-infection).

**Figure-5:**
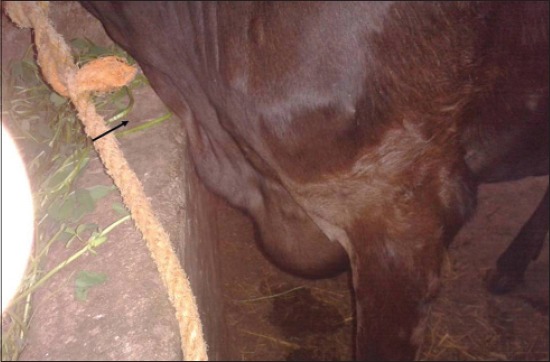
Lumpy skin disease in cow showing edema of ventral abdominal.

**Figure-6:**
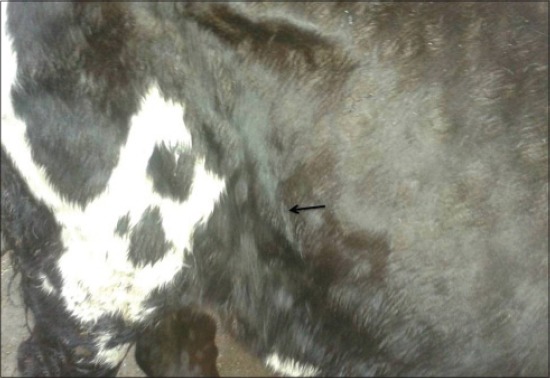
Lumpy skin disease in cow showing enlargement of precrural lymph node.

**Figure-7:**
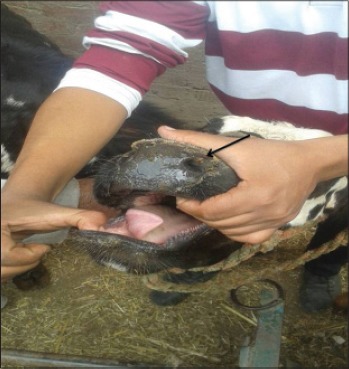
Lumpy skin disease in cow showing nodules and ulcer on upper respiratory.

LSD is a pox viral disease of cattle with a major socio-economic impact [22-24]. The LSD was confirmed by the presence of characteristic pathognomonic [12], eosinophilic intracytoplasmic inclusion bodies in the prickle cell layer (Figure-1) by histopathological examination of surgically removed skin nodules. Recorded clinical signs in this study were in agreement with the previous studies [12,13,25] who mentioned that LSD infected animals showed, pyrexia 40-41°C for large release and rapid clearance of pyrogens [25]. Skin nodules which ranged from a few to several hundred sometimes coalesced together. Later these nodules may be contained a clear serous or purulent exudates furthermore ulcers formation (Figures-2-4). Edema of the ventral abdominal wall and enlargement of superficial lymph nodes could be seen (Figures-5 and 6) [12]. Nasal discharge could be seen as a result of extent of infection to the upper respiratory tract (Figure-7) [7,12].

The innate immune response is the first line of defense of the immune system of the host and is needed for stimulating the adaptive immune response. The cellular components of the innate immune system, namely macrophages and neutrophils are critical for controlling microbial infection. These immune cells destroy the pathogen by phagocytosis and production of reactive oxygen species [26]. In the presented study, immunological parameters revealed a significant decrease of lymphocyte transformation rate, phagocytic activity and killing percentage which was marked within 2 weeks post-infection (Table-1) that may be due to LSD followed by secondary bacterial invasion in addition to stress induced immunosuppression [3].

**Table-1:** Alteration in lymphocyte transformation rate, phagocytic % and killing % in cows naturally infected with LSD virus (mean values±SE).

Groups				

Parameters	Healthy control cows	LSD infected cows				

		Post-infection		F test

		1^st-^2^nd^ day	10-14^th^ days	
LTR	1.378^a^±0.01	1.255^b^±0.01	1.232^b^±0.008	**
Phagocytic %	83.00^a^±0.63	75.80^b^±0.72	73.60^b^±1.20	**
Killing %	80.60^a^±0.50	70.40^b^±0.50	68.40^b^±1.07	**

Means in the same row with different superscript letters are significantly different

** Highly significant difference at P≤0.01. LTR=Lymphocyte transformation rate, LSD=Lumpy skin disease, SE=Standard error

Regarding to the erythrogram results (Table-2) revealed non-significant change in hemogram within 1^st^-2^nd^ day post-infection. At 10-14^th^ day LSD post-infection revealed a significant decrease in red blood cells, hemoglobin, packed cell volume, and mean corpuscular hemoglobin concentration with a significant increase in mean corpuscular volume when compared by healthy control. This result interpreted as a macrocytic hypochromic anemia which in agreement with [27] who reported that hemolytic anemia could be occurred with viral infection. On the other side leucogram results (Table-3) during 1^st^-2^nd^ days post-infection revealed leucopenia and lymphopenia which may be due to viral infection [28]. These results agree with [25] who recorded that a release of large quantities of endogenous corticosteroid could account for a lymphopenia. While 10-14^th^ day post-infection revealed granulocytic leucocytosis which could be due to secondary acute bacterial infections, especially pyogenic bacterial infections [28,29].

**Table-2:** Alteration in erythrogram in cows naturally infected with LSD virus (mean values±SE).

Groups				

Parameters	Healthy control cows	LSD infected cows		

		Post-infection		F test

		1^st-^2^nd^ day	10-14^th^ days	
RBCs count (×10^6^/μl)	7.42^a^±0.11	7.19^a^±0.04	6.65^b^±0.08	**
Hb (g %)	10.9^a^±0.25	10.9^a^±0.07	9.2^b^±0.06	**
PCV (%)	34.20^a^±0.54	34.46^a^±0.22	30.44^b^±0.38	**
MCV (fl)	46.00^b^±0.00	46.00^b^±0.00	48.00^a^±0.00	**
MCH (pg)	14.96^a^±0.15	15.22^a^±0.16	13.76^b^±0.18	**
MCHC (%)	32.52^a^±0.25	31.68^a^±0.33	30.54^b^±0.27	**

Means in the same row with different superscript letters are significantly different.

** Highly significant difference at p≤0.01, *significant difference at P≤0.05. SE=Standard error, PCV=Packed cell volume, RBCs=Red blood cell, MCV=Mean corpuscular volume, MCH=Mean corpuscular hemoglobin, MCHC=Mean corpuscular hemoglobin concentration, LSD=Lumpy skin disease

**Table-3:** Alteration in leucogram in cows naturally infected with LSD virus (mean values±SE).

Groups				

Parameters	Healthy control cows	LSD infected cows		

		Post-infection		F test

		1^st-^2^nd^ day	10-14^th^ days	
WBC count (×10^3^/μl)	9.62^b^±0.14	7.10^c^±0.14	15.82^a^±0.99	**
LYM (×10^3^/μl)	5.60^a^±0.28	3.04^b^±0.24	7.42^a^±1.17	**
MID (×10^3^/μl)	1.39^b^±0.03	0.74^c^±0.05	3.03^a^±0.12	**
GRA (×10^3^/μ)	2.63^b^±0.03	3.32^b^±0.03	5.37^a^±0.03	*

Means in the same row with different superscript letters are significantly different.

** Highly significant difference at p≤0.01

* significant difference at p≤0.05. SE=Standard error, WBC=white blood cell, GRA=Neutrophil, eosinophil and basophil, MID=Monocytes and some eosinophil, MCV=Mean corpuscular volume, MCH=Mean corpuscular hemoglobin, MCHC=Mean corpuscular hemoglobin concentration, LSD=Lumpy skin disease

**Figure-8:**
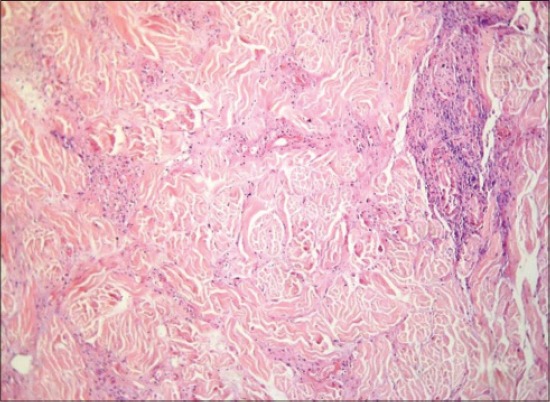
Lumpy skin disease, subcutaneous muscle of cow showing sever coagulative necrosis and calcification, H and E, ×1200.

Regarding to results of biochemical analysis (Table-4) revealed a significant decrease in total protein and albumin, however; there was a significant increased in globulin, especially gamma globulins in LSD infected cows. These results might be attributed to decreased protein synthesis and higher catabolic rate, as well as damaged liver parenchyma [30]. While increased globulin, especially gamma globulins were mainly an immune response following infection [31]. The significant increase of CK in LSD infected cows clearer in late stage could be due to muscle damage involvement [32] that confirmed with histopathological examination which showing sever coagulative necrosis in subcutaneous muscle (Figure-8). So, LSD has economically importance not only induce damaged hides [33], but also involvement of muscle. On the other side, there were highly significant increases in serum activities of ALT and AST in LSD infected cows when compared to healthy control. These results may be due to liver function disturbance [34]. The increase in AST may be also due to the heart muscle and the general tissue breakdown caused by the virus or secondary bacterial infection [35]. Moreover, there was a significant increase in serum creatinine level in LSD cows, this result indicate the direct effect on the kidney by LSDV [28] while a significant increase in blood urea nitrogen in diseased animals in comparison with apparently healthy groups. This could be due to protein breakdown increased in hyperthermia [32].

**Table-4:** Alteration in some biochemical parameters in cows naturally infected with LSD virus (mean values±SE).

Groups				

Parameters	Healthy control	LSD infected cows		

		Post-infection		F test

		1^st-^2^nd^ day	10-14^th^ days	
Total protein (g/dl)	6.95^a^±0.12	6.10^b^±0.10	6.04^b^±0.21	*
Albumin (g/dl)	3.80^a^±0.12	2.40^b^±0.17	2.32^b^±0.10	**
Globulin (g/dl)	3.15^b^±0.18	3.70^a^±0.11	3.72^a^±0.12	*
α globulins (g/dl)	1.11^a^±0.12	1.01^ab^±0.12	0.91^b^±0.12	*
β globulins (g/dl)	0.74^a^±0.12	0.68^a^±0.12	0.73^a^±0.12	NS
γ globulins (g/dl)	1.30^b^±0.12	2.01^a^±0.12	2.08^a^±0.12	**
CKMM (U/L)	240.2^c^±1.88	297.8^b^±2.10	311.4^a^±2.97	**
ALT (U/L)	17.26^b^±0.79	30.54^a^±0.88	30.88^a^±1.23	**
AST (U/L)	47.22^b^±1.35	62.58^a^±1.40	63.12^a^±2.41	**
Creatinine (mg/dl)	0.55^b^±0.03	1.50^a^±0.13	1.47^a^±0.04	**
Urea (mg/dl)	15.4^b^±0.97	21.2^a^±0.58	21.6^a^±0.87	**

Means in the same row with different superscript letters are significantly different.

** Highly significant difference at p≤0.01.

* Significant difference at p≤0.05. NS=Not significant, AST=Aspartate aminotransferase, ALT=Alanine aminotransferase, CKMM=Creatine phosphokinase, LSD=Lumpy skin disease

## Conclusion

LSDV infected cows in early stages revealed leucopenia and immunosuppressive effect, so immunostimulant therapy is required. In late stage revealed hemolytic anemia, leucocytosis and increase of serum CK which could aid in diagnosis. Disturbance in liver and kidney function tests have been occurred.

## Authors’ Contributions

ANFN collected and examined samples, planned the study design, drafted and revised the manuscript and read and approved the final manuscript.
